# Supplementary material for: Identification of Key Aromatic Compounds in Basil (Ocimum L.) Using Sensory Evaluation, Metabolomics and Volatilomics Analysis
Source: Metabolites. 2023 Jan 4;13(1):85. doi: 10.3390/metabo13010085 (PMC9865694; doi:10.3390/metabo13010085)
Supplement: Supplementary file 1 [file Supplemental Figure S1.pdf › 11.28-metabolites-2090276-peer review Supplemental/Supplemental Figure S1.pdf]

Supplemental information

Identification of key aromatic compounds in basil (*Ocimum* L.) using sensory evaluation, metabolomics and volatilomics analysis

Pengmeng Du, Honglun Yuan, Yayu Chen, Haihong Zhou, Youjin Zhang, Menglan Huang, Yiding Jiangfang, Rongxiu Su, Qiyu Chen, Jun Lai, Lingliang Guan, Yuanhao Ding, Haiyan Hu and Jie Luo

Document S1. Supplemental Figures S1

Supplemental Figure S1 Correlation analysis between sensory evaluation results and metabolome/volatilome data

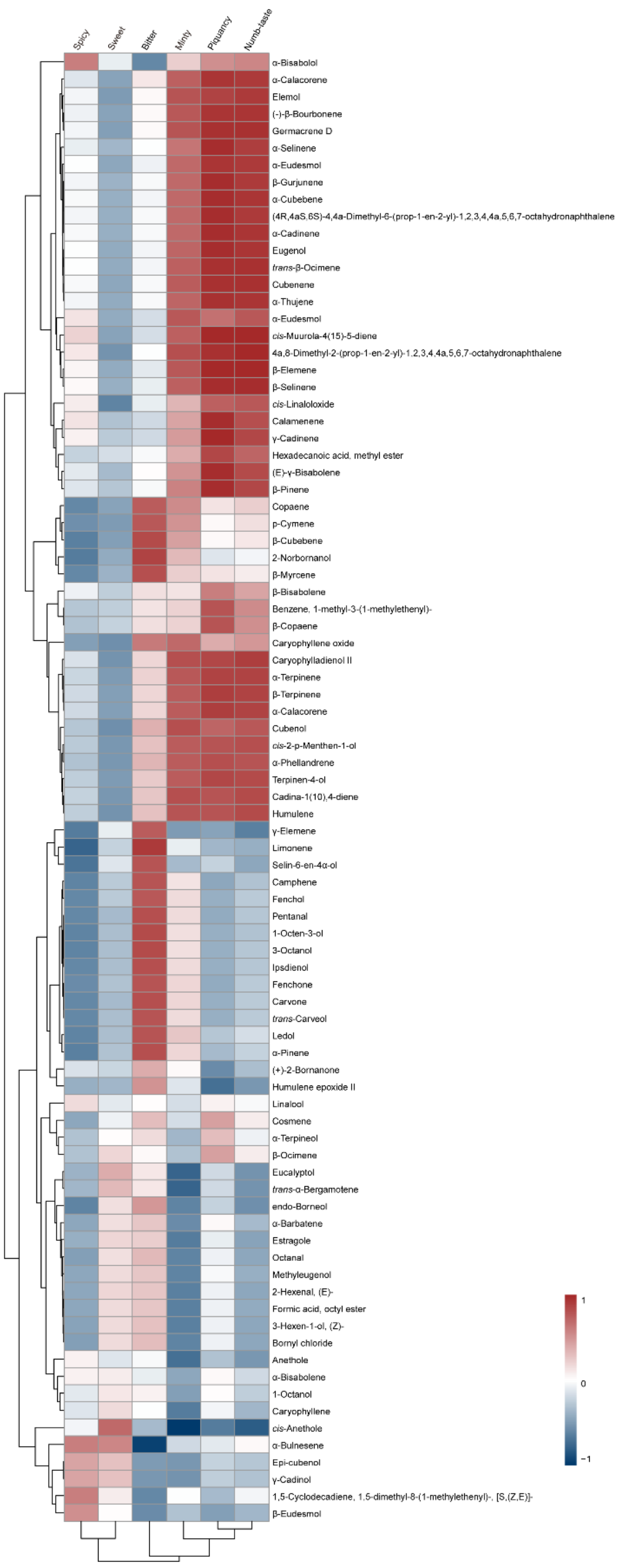

Supplemental Figure S1 Correlation analysis between sensory evaluation results and metabolome/volatilome data. Correlation analysis between different attributes rating in retronasal evaluation and volatilome data. Red and blue indicates positive and negative correlation.
